# Role of End Plate Changes and Paraspinal Muscle Pathology in Lower Back Pain: A Narrative Review

**DOI:** 10.7759/cureus.61319

**Published:** 2024-05-29

**Authors:** Manasa Suryadevara, Gaurav V Mishra, Pratapsingh Parihar, Chaitanya Kumar Javvaji, Anshul Sood, Harshitha Reddy, Naramreddy sudheesh Reddy, Sheetal S Shelar

**Affiliations:** 1 Radiodiagnosis, Datta Meghe Institute of Higher Education and Research, Wardha, IND; 2 Pediatrics, Datta Meghe Institute of Higher Education and Research, Wardha, IND; 3 Internal Medicine, Datta Meghe Institute of Higher Education and Research, Wardha, IND

**Keywords:** end plate, mri imaging, lower back pain (lbp), modic changes, paraspinal muscles

## Abstract

Degenerative changes of the lumbar intervertebral disc are the most significant causes of enduring lower back pain. The possibility of the diagnosis is limited in people with this low back pain. Therefore, it is essential to identify the relevant back pain subgroups. The paraspinal muscles, that is, the muscles that attach to the spine, are necessary for the proper functioning of the spine and the body; insufficiency can result in back pain. Lower back pain disorders are strongly associated with altered function or structure of these paraspinal muscles, especially fibrosis and fatty infiltration. Modic changes are the bone marrow changes of the end plate in the vertebral body seen on MRI. These are strongly related to degeneration of the disc and are common in individuals with back pain symptoms. Articles were selected from Google Scholar using the terms 'Modic changes,' 'end plate changes,' 'paraspinal muscles,' and 'lower back pain. ' This article compiled different studies aiming to enhance the comprehension of biochemical processes resulting in the development of lumbar pain. Search using the keywords 'Modic changes,'' end plate changes lower back pain,' 'paraspinal muscles lower back pain,' and 'Modic changes lower back pain' on Google Scholar yielded 33000, 41000, 49400, and 17,800 results, and 958, 118, 890 and 560 results on Pubmed respectively.

## Introduction and background

Chronic lower back pain is still a challenging clinical issue, both in terms of treatment and also diagnosis. Despite the prevailing view that back pain is mainly annulogenic, which arises due to the sensitization of pain receptors present within the annulus fibrosus (outer layer of the intervertebral disc) of degenerating discs, there is rising evidence that the damage in end plates may signify a painful pathology (vertebrogenic pain) [1]. It is most likely for the reason that endplate defects are areas of structural vulnerability that could amplify the interaction of proinflammatory elements between the vertebra and disc leading to chemical sensitization and bone marrow neo-innervation [2].

The paraspinal muscles (multifidus, psoas major, erector spinae) are critical for the functioning and stability of the spine [3]. Fat replacement and muscle atrophy are characteristic features of discogenic low back pain (DLBP). The remodelling and fat infiltration of the paraspinal muscles may deteriorate DLBP [4]. Fatty infiltration of these muscles may worsen back pain, considering the significant role of paraspinal muscles (PSM) on the lumbar spine. Hence, assessing the association of atrophy and the infiltration of fat in the paraspinal muscles with DLBP is crucial. For patients diagnosed with lumbar degeneration, assessment of the PSM via MRI has been suggested as a marker [5,6]. MRI is broadly utilized in evaluating individuals with lower back pain. MRI primarily offers structural data of the soft tissues, including the water and fat content through the T1 and T2 sequences. Assessing via T1 and T2 imaging mainly focuses on the reactive changes in vertebral bone marrow, structural alterations in the intervertebral disk, extent of displacement of disc material, degree of the stenosis, site, and degree of compression of nerve roots [7].

## Review

Methodology

We searched Google Scholar and PubMed for studies reporting Modic changes and other pathologies like paraspinal fatty infiltration and muscle atrophy that could result in lower back pain. Articles chosen for this review have been searched from accredited sites such as Google Scholar and PubMed. The keywords used were 'lower back pain,' 'end plate changes,' and paraspinal muscles.' We mainly used articles that were published in the international literature.

Review of literature

It is still challenging for clinicians to diagnose patients with lower back pain. It is commonly specified that only a minor percentage (around 20%) of patients with low back pain (LBP) can be diagnosed with reasonable certainty [8]. Differentiating amongst types of this pain will probably be crucial for improving patient outcomes since a proper identification of the cause of the pain is required for ideal management [1].

The end plate comprises a double layer of bone and cartilage, which separates intervertebral discs and the adjacent vertebrae [9]. In certain individuals, there is another additional dense bone layer beneath the outermost layer [10,11]. The end plate, through its structure, aids essential nutritional and biomechanical functions. This end plate is biomechanically prone to momentous pressure during daily events, particularly when trunk muscles contract to maintain the stability of posture [1]. The end plates are involved in distributing this intradiscal pressure onto the vertebrae adjacent to them and preventing the nucleus of the disc from protruding into the underlying trabecular bone [12-14]. Between the pliable intervertebral disks and rigid vertebral bodies, the end plates serve as the junction. Since the spine carries momentous pressure and the discs do not have a devoted blood supply, these end plates should be porous to enable transport between the capillaries of vertebrae and disk cells and also be strong to prevent vertebral fracture. Consequently, end plates are notably prone to injury, which may enhance communication among vascularized vertebral bone marrow and proinflammatory components of the disc. Areas of damaged end plates can lead to the formation of reactive bone marrow lesions, often accompanied by nerve proliferation, rendering them susceptible to mechanical stimulation and chemical sensitization [1]. Many consider provocation diskography (PD) the most specific method for diagnosing disc-related pain [15]. The association between abnormalities in the vertebral bone marrow and PD-confirmed discogenic pain is possibly the most substantial evidence of end plate involvement in lower back pain.

Three types of signal changes in the marrow of the vertebra next to the end plate were described in 1988 by Modic et al. [16,17]. Modic changes type I show a high signal on T2, appear hypointense on T1 images, and represent fibrovascular replacement. Histopathological examination of Modic type I changes reveals a phase of active inflammation, which corresponds to the end plate fissuring and disruption, alongside the presence of vascularized granulation tissue within the marrow. Type II Modic changes display a high signal intensity on both T2 and T1 sequences, correlating with fat replacement in the marrow. Both these types exhibit dynamic characteristics, as the type I changes can either convert back to normal marrow or to type II changes, and likewise, the type II may change back to type I changes [18-20].

Conversely, the Modic changes type III seem hypointense on both T2-weighted imaging (T2WI) and T1-weighted imaging, representing sclerosis. Information from various studies indicates that type I and II Modic changes stand out as highly specific MRI observations in predicting concurring pain induced by PD [1]. In a study, the presence of severe Modic type I or II end plate defects consistently correlated with positive concurring pain at the adjacent disc, showing a correlation rate of 100% [21]. Recent studies have reported that in end plates with Modic changes, increased end plate innervation was noted. However, Modic changes are not highly sensitive to PD-confirmed disc pain. This poor sensitivity may result from the subjective methods used to classify bone marrow lesions (BML) rather than quantitative and objective techniques. Additionally, standard MRI techniques may not effectively visualize innervated end plate damage due to the end plate's short T2, which produces a minimal signal with pulse sequences that have longer echo times. Newer imaging sequences with ultrashort echo times could potentially improve the differentiation between patients with and without end plate pathologies [1].

Endplate damage serves as a disposing factor for the communication between the marrow of vertebrae and the nucleus and has been qualitatively related to bone marrow lesions containing pain fibers [22,23], and more directly, related to the markedly increased diffusion between the nucleus and vertebra [24,25]. Thus, various types of end plate abnormalities have been linked with axial back pain and disk degeneration clinically [26-29]. These comprise calcifications, avulsions/erosions, Schmorl nodes, and fractures. Schmorl nodes are prominent, focal indentations of the end plate, representing the nucleus herniating into vertebrae adjacent to them, and are linked with the disc degeneration severity significantly [30]. In a recent study relating various forms of end plate abnormalities in patients with lower back pain history, a distinct clear dose effect was noticed, demonstrating that the bigger lesions were linked with a higher incidence of back pain and a more severe degeneration [31].

The PSM, which limit excessive intervertebral movement and stabilize posture, are another important mediator of pain [32,33]. Paraspinal muscles (erector spinae, multifidus, psoas major) play a vital role in maintaining the functioning of the lumbar spine and its stability [3]. Fat infiltration and muscle atrophy are considered to be the features of DLBP. This fat infiltration and paraspinal muscle remodelling can worsen the pain [4]. Several studies proved a strong association between low back pain and degeneration of paraspinal muscle. Poor functioning PSM can affect the alignment of the lumbar spine [34] and impair the spinal biomechanics [35], which, in turn, increases microtrauma potential and disc stress [36,37]. It is, therefore, not surprising that lower back pain and related disability are thought to be linked to poor PSM function and quality (lean muscle and relative amounts of fat seen on the MRI).

Previous studies have shown low CT attenuation and bright signal on T2WI in the PSM of lower back pain patients due to infiltration of fat [38]. The arrival of MRI has brought significant focus to the complex relationship between the body of vertebra, intervertebral discs, facet joints, and end plates, also their roles in the pathogenesis of back pain [39]. Recently, advanced chemical shift-encoded MRI (CSE-MRI) was proposed for fat assessment quantitatively in different parts of the body non-invasively. Through the CSE-MRI, the proton density fat fraction (PDFF) assessed is accurate and has high repeatability in determining muscle fat composition quantitatively. Among the CSE-MRI techniques, IDEAL-IQ (iterative decomposition of water and fat with echo asymmetry and least-squares estimation) stands out and is comparable to MR spectroscopy for assessing fat infiltration of muscle. Moreover, IDEAL IQ and other CSE-MRI techniques have noteworthy advantages over MRS, like quick imaging of anatomical structures and volumetric data acquisition. Hence, PDFF aids in quantifying and clarifying the fatty infiltration within the PSM of patients with discogenic low back pain [38].

Chen et al. [40], in a study involving 153 patients who took lumbar MRI scan for low back pain from 2015 to 2017, showed type II Modic changes in 71.9% of patients, followed by type I Modic changes in 22.9% of patients and type III changes in 5.2% of patients, concluding that type II changes are most common in people with lower back pain. Patients under 50 likely developed type I Modic changes, while those with age over 50 likely had type II changes. The disc level most involved was L5-S1, followed by the L4-L5 level.

Chen et al. [41], in a study done in 2019, reported that end plate abnormalities have been linked with lower back pain and present an apparent dosage effect due to their strong association with degeneration of the adjacent disc. These severe end plate defects lead to poor efficacy, lead to inadequate nutrition of the disc, and damage to the lumbar stability.

Kjaer et al. [42], through a study done in 2001 involving a group of 412 persons aged around 40 years from the general population, demonstrated a strong association with lower back pain through the one year of study, predominantly for Modic change type 1. The study showed that the pain developed in 88% of people with Modic changes and only 12% in people without Modic changes within the last year.

Albert and Manniche [43], in a study done during 2005-2006, evaluated 166 patients with sciatica, where all the patients had undergone MRI in the acute period and also after a 14-month period. After the follow-up, it was noticed that around 60% of patients with Modic changes suffered from lower back pain. In contrast, only 20% had LBP in the group without Modic changes, and in these 14 months, the occurrence of Modic changes increased to 24%, i.e., from 25% to 49%. Compared to people with type 2 Modic changes, in people with type I changes, the pain was more frequent, though not very significant statistically. Kjaer et al. [42] and Toyone et al. [44] also observed similar findings. These studies thus showed that compared to the type II changes, the type 1 Modic changes are possibly more associated with pain. The fact that Modic changes type 1 changes represent an earlier and more active inflammatory phase could be the reason that they are more painful.

Braithwaite [45], through a study in 1997 involving 58 individuals with discogenic back pain who were advised provocative discography before the fusion, demonstrated that around 48% of these patients showed the presence of Modic changes, especially type II. It was also revealed that Modic change is a definite indicator of discogenic pain with a specificity of 0.97. However, the sensitivity was low, that is, the lower back pain can also be due to other causes. In other studies, the Modic changes have also been revealed to be painful in discography.

Ohtori [23], during surgery in 2005, evaluated endplates of 14 patients who presented with lower back pain showing Modic changes and four controls without Modic changes. Following preparation, it was found that about 86% of the patients with Modic changes exhibited the presence of PGP 9.5 immunoreactive nerve fibers in the cartilage of the endplate, as opposed to none of the controls. Also, in both patients and controls, endplates showed the presence of tumor necrosis factor (TNF) immunoreactivity, but compared to controls, patients with Modic changes showed markedly higher cytokine levels and, in particular, significantly more in patients with type I Modic changes. Regardless of whether the patient presented with Modic changes or not, the bone marrow of vertebrae showed only a few PGP 9.5 immunoreactive fibers and showed no presence of TNF immunoreactivity. The presence of Substance P immunoreactive fibers and these pro-inflammatory cytokines may explain why the Modic changes cause pain [23]. In the majority of the cases, since Modic changes, are detected in concurrence with disc degeneration, the idea of ''internal disc disruption'' has been proposed by Crock [46]. According to this theory, inflammatory substances could be produced in the nucleus pulposus due to repetitive disc trauma. They can result in an inflammatory reaction locally when these toxic inflammatory chemicals diffuse through the endplates, resulting in back pain.

Through a study, Crawford et al. [47] discovered that asymptomatic females demonstrated more fatty infiltration and smaller cross-sectional area (CSA) of the multifidus than asymptomatic males. Sasaki et al. observed that males exhibited larger CSA and lower fat infiltration percentage (FI%) in the paraspinal muscles compared to females [48]. It was noticed that the lumbar disc degeneration (LDD) patients continued to exhibit these sex-dependent differences. Kim et al. [49] studied a group of 100,000 people and established that the occurrence of herniation of lumbar disc was higher in females compared to males, which is potentially attributed to the sex-dependent disparity in the paraspinal muscle morphology.

Ding et al. [50], in a study done in 2022 involving 76 patients with degenerative lumbar kyphosis (DLK) and 78 patients with degenerative lumbar spondylolisthesis (DLS) measured paraspinal muscle parameters, observed that the atrophy of multifidus is significant in patients with DLS and that DLS patients exhibited greater fat infiltration in the multifidus.

Thakar et al. in 2014 demonstrated through a study that segmental atrophy of multifidus can be seen in patients with DLS, and there can be compensatory hypertrophy of erector spinae muscle [51].

Wan et al. [52], through a study involving 178 patients with one-sided lower back pain who underwent lumbar MRI, measured the cross-sectional areas and mean signal intensities of PSM bilaterally and calculated the fat infiltration percentage. It was noticed that the acute pain group showed markedly reduced CSA of the psoas and erector muscles, while there was a substantial reduction of CSA of the multifidus and erector muscles in the chronic pain group, especially on the painful side. The fat content of erector muscle was markedly larger in the chronic pain group compared to the acute pain group, particularly on the painful side, there was a significant reduction of CSA in multifidus muscle at many levels.

Mengiardi et al. [53] included 25 patients with chronic lower back pain and 25 asymptomatic volunteers as the control in a study and observed that patients with chronic lower back pain (CLBP) had a substantially greater fat component percentage in the multifidus muscle compared to the asymptomatic individuals. Compared to the asymptomatic individuals, in patients with CLBP, Proton MRS reveals a larger fat component in the multifidus.

Chan et al. [54], through a study involving 12 patients with CLBP and 12 asymptomatic individuals as controls, demonstrated that posture and lower back pain are linked with changes in both CSA and elasticity of the multifidus muscle. The muscle was smaller, stiffer, and had greater fat component in patients with CLBP compared to the asymptomatic individuals.

Kjaer et al. [4] involved 442 adolescents and 412 adults from the general population in a cohort study, and using MRI, visually graded the fatty infiltration of multifidus muscle as severe, slight, or none. The odd ratio was calculated for both age groups and concluded that 81% of adults showed fat infiltration while fatty infiltration was noted only in 14% of the adolescents. The study showed severe fatty infiltration in the adults and that this fat infiltration was strongly linked with lower back pain.

Kader et al. [55] studied 90 patients having either low back pain with leg pain or mechanical low back pain for three months. Patients with a structural deformity, previous surgery, infection, tumour, or spinal fracture were excluded. The MR images of 75 individuals were analyzed, and the level and side of multifidus muscle degeneration were identified by a reduction in the muscle size. The muscle atrophy was graded as severe, moderate, and mild. Significant degeneration of multifidus muscle was noticed in about 80% of patients with lower back pain. It was mostly bilateral and mainly demonstrated at L4-L5 and L5-S1 levels. The atrophy of muscle was relatively common in female patients and the elderly. The significant correlation between leg pain and the multifidus muscle atrophy was the most important finding.

Kang et al. [56], through a study comprising patients with DLK and a control group with lower back pain, compared the lumbar musculature of both groups and concluded that the individuals with LDK exhibit reduced CSAs of the psoas, erector spinae, and multifidus muscles compared to CLBP patients without DLK. Moreover, DLK patients demonstrate diminished lumbar muscularity compared to the control group. The ratios of psoas-to-disc, erector spinae-to-disc, and multifidus-to-disc were all notably lesser in the DLK group compared to the control group. These results add to the growing evidence suggesting that the CSA of paraspinal muscles is reduced in individuals with postoperative low back pain and CLBP. In patients with DLK, the multifidus muscle showed more specific muscle wasting than in the patients with CLBP.

The results of the review are listed in Table 1.

**Table 1:** Population data table. MC, Modic changes. CSA, cross-sectional area. DLK, degenerative lumbar kyphosis. DLS, degenerative lumbar spondylolisthesis. CLBP, chronic lower back pain

Reference	Number of subjects	Conclusion
Kjaer et al [42]	412 subjects (199 males and 213 females)	The general population aged over 40 has a strong association with LBP, particularly for type 1 Modic changes. Type I Modic changes are more frequent compared to type 2 changes.
Albert and Manniche [43]	166 patients with sciatica	60% of patients with Modic changes suffered from lower back pain, while pain prevailed only in 20% of patients without Modic changes.
Braithwaite [45]	58 patients with discogenic pain	48% of patients showed the presence of MC, predominantly type 2. Modic changes are highly specific to painful discs.
Yufeng Chen et al. [40]	153 patients	Type II MC were the most common, followed by type I and they mostly occur at L4-L5 and L5-S1 levels
Yufeng Chen et al. [41]	128 patients with endplate changes following discectomy	Severe endplate changes damaged spine stability and resulted in chronic lower back pain. Discectomy had a noticeable efficacy.
Ohtori [23]	14 patients with LBP and 4 controls	Noticed end plates during surgery that 86% of patients had immunoreactive nerve fibers in the cartilage, and these cytokines were comparatively higher in patients with MC.
Crawford et al. [47]	80 healthy volunteers	The fat component in lumbar multifidus and erector spinae increased with age, was higher in females than males, and is more prevalent in multifidus.
Thakar et al. [51]	120 patients	Segmental multifidus atrophy is seen in patients with DLS. Whereas their erector spinae undergo compensatory hypertrophy. Female gender predisposes to decreased psoas muscle area.
Jun‑zhe Ding et al. [50]	154 patients (78 patients with DLS and 76 patients with DLK)	Multifidus degeneration is more significant in the spondylolisthesis group and erector spinae in kyphosis. Infiltration of fat in the lumbar multifidus was higher in the DLS patients as compared to that of DLK patients.
Qing Wan et al. [52]	178 patients with one-sided lower back pain who underwent MRI	The cross-sectional areas of the erector and psoas muscles were decreased in the acute pain group significantly whereas the chronic pain group showed a significant decrease of CSA of the erector and multifidus muscles especially on the painful side. The fat content of erector muscle was significantly higher on the painful side in the chronic pain group compared to the acute pain group, and also, on the painful side, there was a significant reduction of CSA in multifidus muscle at various levels.
Bernard Mengiardi et al. [53]	25 patients with CLBP and 25 asymptomatic individuals (13 females and 12 males)	Patients with CLBP had a bigger fat percentage in the multifidus muscle when compared to the asymptomatic individuals.
Suk-Tak Chan et al. [54]	12 adult males with chronic LBP and 12 male controls.	Lower back pain and posture are linked with changes in CSA and elasticity of the multifidus. The muscle was smaller, stiffer, and had a higher fat component in patients with CLBP compared to the asymptomatic controls.
Per Kjaer et al. [4]	442 adolescents and 412 adults	81% of adults showed fat infiltration, while fatty infiltration was noted only in 14% of the adolescents. The study showed that there was severe fatty infiltration in the adults and that this fat infiltration was strongly linked with lower back pain while no such association was noted in the adolescents.
D. F. Kader et al. [55]	90 patients having either low back pain with leg pain or mechanical lower back pain for 3 months	Significant degeneration of multifidus muscle was noticed in about 80% of patients with lower back pain. It was mostly bilateral and mainly demonstrated at L4-L5 and L5-S1 levels. The atrophy of muscle was relatively common in female patients and the elderly. The most important finding was the significant correlation between leg pain and the multifidus muscle atrophy.
C.H. Kang et al. [56]	54 patients having degenerative lumbar kyphosis (DLK) and a control group of 54 individuals with lower back pain	CSAs of the multifidus, the psoas, and the erector spinae muscles in patients with DLK are reduced compared to patients with CLBP without lumbar kyphosis and show that patients with DLK have lesser lumbar muscularity compared to the control group.

## Conclusions

This article aims to review the data on endplate abnormalities and evidence for the role of pathologic changes involving end plates and paraspinal muscles as a cause of chronic low back pain. This review paper discussed the role of Modic changes and the quality of paraspinal muscles in lower back pain. This article aimed to show the association between abnormalities of endplate and decreased PSM quality, including relatively poor quality of PSM being predictive of CLBP. Endplate defects are associated with back pain and are strongly related to the degeneration of adjacent discs, with an apparent dosage effect. Different magnitudes of disc degeneration seem to be associated with varying types of endplate defects. Lesions in the lumbar endplate may be crucial in better understanding back pain and degeneration of the disc.

This study compared patients with Modic and endplate changes to healthy controls, which displayed the prevalence of fatty degeneration of lumbar paraspinal muscle and showed that compared to healthy controls. Individuals with Modic changes have smaller cross-sectional areas but more significant fatty infiltration.
